# Assessing the Demand for Plastic Latrine Slabs in Rural Kenya

**DOI:** 10.4269/ajtmh.18-0888

**Published:** 2019-08-05

**Authors:** Rachel Peletz, Joyce Kisiangani, Patrick Ronoh, Alicea Cock-Esteb, Claire Chase, Ranjiv Khush, Jill Luoto

**Affiliations:** 1 Aquaya Institute, San Anselmo, California;; 2 Aquaya Institute, Nairobi, Kenya;; 3 World Bank, Washington, District of Columbia;; 4 Rand Corporation, Santa Monica, California

## Abstract

Improving access to safe and affordable sanitation facilities is a global health priority that is essential for meeting the United Nation’s Sustainable Development Goals. To promote the use of improved sanitation in rural and low-income settings, plastic latrine slabs provide a simple option for upgrading traditional pit latrines. The International Finance Corporation/World Bank Selling Sanitation program estimated that plastic slabs would have a 34% annual growth, with a market size of US$2.53 million in Kenya by 2017. In this study, we examined the commercial viability of these plastic latrine slabs in rural Kenya by evaluating a financing and distribution model intervention, documenting household slab sales to date, and assessing consumer exposure and perceptions. We also determined household willingness to pay through a real-money auction with 322 households. We found that no households in our study area had purchased the plastic slabs. The primary barriers to slab sales were limited marketing activities and low demand compared with the sales price: households were willing to pay an average of US$5 compared with a market price of US$16. Therefore, current household demand for the plastic latrine slabs in rural Kenya is too low to support commercial distribution. Further efforts are required to align the price of plastic latrine slabs with consumer demand in this setting, such as additional demand creation, product financing, and public sector investment.

## INTRODUCTION

Globally, more than 2.3 billion people lacked access to basic sanitation services in 2015.^1^ Poor sanitation is associated with diarrhea, helminth infection, and other infectious diseases,^2,3^ in addition to environmental enteric dysfunction^4^ and child growth faltering.^5^ Notwithstanding challenges in quantifying the health impacts of sanitation interventions,^6^ unsafe sanitation is estimated to cause almost 900,000 deaths every year.^7^ This health and economic burden is disproportionally borne by the poor. In Kenya alone, the World Bank estimates that limited access to sanitation costs US$324 million in lost productivity, equivalent to nearly 1% of annual gross domestic product.^8^

In rural Kenya, approximately 85% of households use simple pit latrines.^1^ Most of these pit latrines, however, do not meet the WHO/UNICEF Joint Monitoring Program (JMP) specifications for improved sanitation facilities: only 42% of Kenya’s rural population uses improved latrines (including shared facilities).^1^ Accelerated improvements in sanitation are needed for Kenya to meet the Sustainable Development Goal target 6.2, which specifies adequate and equitable sanitation for all by 2030.^9^

Latrine slabs are a potential simple option for upgrading pit latrines. By providing a smooth, easily cleaned, and safe squat hole opening, latrine slabs comply with the WHO/UNICEF JMP and Government of Kenya definitions for improved sanitation facilities.^1,10^ The Kenya Environmental Sanitation and Hygiene Policy for 2016–2030 specifically identifies latrine slabs as an appropriate technology for improving traditional pit latrines.^10^ Although some latrine slab options exist in the Kenyan market, ready-made products are not available to rural households: ceramic pans generally target urban consumers as part of flush toilet solutions; precast concrete slabs are not common, possibly because of the high transport costs; and existing plastic latrine slabs are generally produced for the donor community for emergency relief and other aid-related operations.^11^ Other alternatives include poured concrete slabs, which usually require a local mason to make and install the slab on-site, although they are often prohibitively expensive (costing US$50–US$60 in Kenya).^11,12^ Unimproved options, such as locally sourced mud, wood, or other materials, are common, but the less rigid structures can be unsafe and difficult to keep clean.^12^

**Figure 1.**
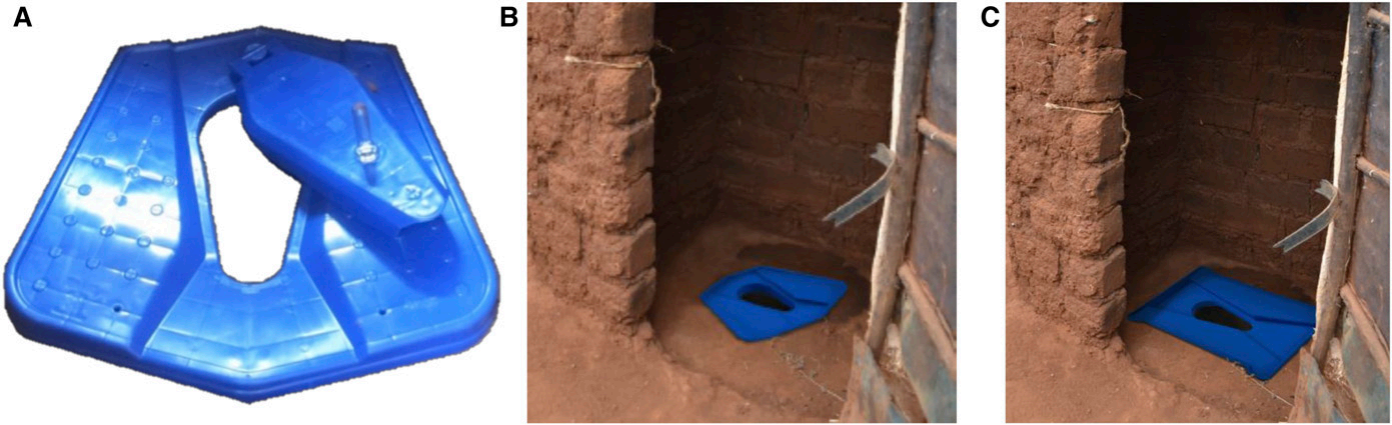
Plastic latrine slab product, manufactured by SilAfrica: (**A**) small plastic slab, 60 × 60 cm, (**B**) small plastic slab installed in pit latrine, and (**C**) larger plastic slab, 80 × 60 cm, installed in a pit latrine. This figure appears in color at www.ajtmh.org.

As an affordable, commercially available, alternative to improve sanitation at a lower cost, the Selling Sanitation program collaborated with large plastics manufacturing firms in Nairobi to develop a range of new plastic slab products (Figure 1).^12,13^ Managed by the former Water and Sanitation Program (WSP) of the World Bank (relaunched as the Global Water Security & Sanitation Partnership in 2017) and the International Finance Corporation (IFC), this program included extensive market research, consumer interviews, and product testing to incorporate consumer preferences and behaviors into the design of the product.^11^ The resulting plastic latrine slabs were designed to be low cost, durable, easy to clean, and lightweight for transport; in addition, they included a foot-operated drop-hole cover to minimize flies and odors (Figure 1). To date, WSP, IFC, private sector, and the Bill & Melinda Gates Foundation have collectively invested approximately US$2 million in the development, testing, and market development support of the plastic latrine slabs (Claire Chase, personal communication).

In 2013, the Selling Sanitation program estimated that the sanitation market in rural Kenya would reach more than two million consumers with 5.8 billion Kenyan shillings (KES) (US$57 million) in revenue by 2017.^14^ This rapid growth assumed both the introduction of new improved latrine products to first-time latrine owners and renovations to existing latrines (almost one million first-time latrine owners and approximately 1.3 million new household consumers for renovation of existing latrines by 2017).^14^ Specifically, the program estimated that plastic slab products would have 34% annual growth, with a market size of 258 million KES (US$2.53 million) by 2017.^14^ These projections presumed that removing “first-mover barriers” (e.g., cost of redesigning products for consumer market, understanding market needs, and linking up with behavior change promotion activities) with initial investments would catalyze sales to the consumer market.^14,15^

This is the first study to examine actual uptake of the new plastic latrine slab in rural households in Kenya. We evaluated the effectiveness of distribution and financing mechanisms that linked a microfinance institution (MFI) with community-based organizations (CBOs) and community leaders by studying sales to households, consumer exposure and perceptions, and household demand measured in terms of willingness to pay (WTP).

## METHODS

### Study design and study population.

#### Study design.

This study was nested within a three-arm cluster-randomized controlled trial (cRCT) with random assignment at village (cluster) level, described elsewhere.^13^ The study stratified villages by county and randomly selected a total of 30 villages (from village lists provided by County Health Offices) to receive the slab distribution and financing intervention. The cRCT aimed to evaluate the effectiveness of engaging CBOs linked with a MFI for plastic slab marketing and distribution to low-income rural households, and compare slab uptake between this arm and a control arm; however, because of low overall sales reported by the slab manufacturers themselves, we did not collect follow-up data in the control arm and did not conduct comparisons between the intervention and control groups.

As a result of low uptake of the plastic slabs, we modified follow-up data collection to adopt a mixed methods approach to evaluate the financing and distribution model intervention arm, including an endline survey with households, real-money auctions with households to determine slab WTP, questionnaires and focus group discussions (FGDs) with CBOs and community leaders, and in-depth interviews (IDIs) with key stakeholders. We conducted our research in 30 villages across Busia and Nyeri counties in Kenya from March 2015 to April 2017. These counties were selected because of their exposure to the pilot improved sanitation campaign (see section Improved Sanitation Campaign).

#### Participant selection and sampling.

We first mapped all households in the study village by visiting every household within the village boundary to ask a short set of questions for household identification and to assess study eligibility through latrine observation. Households were eligible to participate if 1) they had an unimproved pit latrine (defined as having a mud, dirt, or wood floor) and 2) their pit latrine was not full (contents were at least 0.5 m from the latrine floor). Then, using a computerized random number generator, we randomly selected eligible households to participate in the baseline survey (15 households per village). Enumeration teams revisited households the day after village mapping to confirm eligibility and conduct the baseline survey.

Community health volunteers (CHVs) recruited CBOs and community leaders who had the potential to encourage financial products and savings for the plastic slab.^16^ Approximately 15 individuals were recruited per village and 60% were members of groups that engaged in group banking, providing loans, or other financial activities (such as Savings and Credit Cooperatives, or micro-savings groups called *Chamas*). In addition to CBO members, these individuals included village elders/chiefs (community leaders who led local forums where the plastic slab could potentially be promoted), CHVs (established, trusted sources of health information in the community), and masons (known as *fundis*, who could present sanitation options to households and provide technical support in slab installation).

We conducted IDIs with plastic slab manufacturers (SilAfrica Ltd., Nairobi, Kenya and Kentainers Ltd., Nairobi, Kenya), the MFI Ecumenical Church Loan Fund (ECLOF), the slab distributor Fargo Courier, slab retail outlets, and WSP staff members.

### Financing and distribution model intervention.

The financing and distribution intervention was designed to support CBOs and community leaders in serving as the “last mile” link between the plastic slab manufacturers, the MFI (ECLOF), retailers, and households.^13^ Specifically, this program targeted CBOs, village elders/chiefs, CHVs, and masons to be sales representatives who would market the slabs; we refer to these individuals as “sales representatives.” The program intended for these sales representatives to profit from purchasing the slabs in bulk through SilAfrica or ECLOF to then sell in their communities (Table 1). Consumers had three options for purchasing the plastic latrine slabs: from retail shops, from the manufacturer SilAfrica, and from the MFI (ECLOF) (Table 1). We informed the sales representatives of these options during the village trainings. Further financing and distribution model intervention activities consisted of 1) the improved sanitation campaign, 2) village trainings of CBOs and community leaders, and 3) text messages sent to CBOs and community leaders.

**Table 1:** Slab purchase options

Purchase Option	Conditions	Price
Retail shops	1 retailer in Busia town, 1 in Nyeri town	Small slab in Nyeri = 2,500 KES (US$24.5)
	Cash purchase upfront and in-person	Small slab in Busia = 2,000 KES (US$19.6)
	Customers to arrange transport to and from village (9 to 33 km away)	Medium slab = 3,000 KES (US$29.4) at both retailers

SilAfrica (manufacturer)	Customers were required to purchase 3 or 10 packs (slabs could not be sold individually)	Small slab 10 pack = 2,200 KES (US$21.6) per slab
	Mobile phone payments (M-Pesa) required before delivery	Small slab 3 pack = 2,500 KES (US$24.5) per slab.
	Fargo Courier delivered slabs to Busia/Nyeri towns; customers to arrange transport to villages (9–33 km away)	Medium slab 10 pack = 3,700 KES (US$36.3)
		Medium slab 3 pack = 4,000 KES (US$39.2).

ECLOF (MFI)	Only group loans (not individual)	Same as via SilAfrica, with additional interest:
	Requirements	11% interest (and 6 month repayment) for loans ≤ 50,000 KES [US$490]
	1) Form a group of at least ten people	22% interest per year (and 3 year repayment) for loans > 50,000 [US$490]
	2) Register with ECLOF using their personal identification cards	
	3) Deposit 10% of the loan over five weeks (or a minimum of 200 KES [US$2] per person per week for five weeks, for a minimum deposit of 10,000 KES [US$98])	
	4) Meet as a group at least five times over five weeks.	
	After loan approval, ECLOF would pay SilAfrica for the slabs, and SilAfrica would send the slabs to ECLOF who then distributed them to consumers.	

ECLOF = Ecumenical Church Loan Fund; MFI = microfinance institution.

#### Improved sanitation campaign.

All villages participated in the “*My Toilet*, *My Dignity*” improved sanitation campaign ongoing in Busia and Nyeri counties from April–July 2015. Funded by the World Bank, this campaign supported the Kenyan Ministry of Health’s efforts to increase access to improved sanitation in areas where community-led total sanitation triggering activities had already taken place. Implemented by Population Services Kenya in partnership with Population Services International, Experimental Momentum Limited, and Ministry of Health Kenya, the campaign included radio promotion, road shows, posters, and training of CHVs and public health officers to promote sanitation in household visits and small group sessions.^13,17^ The campaign promoted the plastic slab as a sanitation solution that was durable, easy to clean, portable, and easy to install. We examined the extent to which households were exposed to the sanitation campaign through the household endline surveys.

#### Village trainings.

After completing the baseline survey, our enumeration teams conducted village training sessions to promote improved sanitation and introduce the plastic slab to the sales representatives. The half-day trainings included information on the sanitation campaign, product features and benefits, an installation demonstration, options for obtaining the slabs from MFIs or manufacturers, and financing options. These training sessions also introduced the plastic slabs as a potential business opportunity for participants; slabs could be purchased in bulk and financed with credit via the MFI (ECLOF) (detailed in Table 1). In total, we conducted 29 trainings (covering 30 villages, two villages were combined), with an average of 14 participants in each (396 sales representatives in total).

Sales representatives filled out pretraining questionnaires to provide details on their CBOs’ activities (e.g., group banking and providing loans) and their own sanitation facilities. They also filled out post-training questionnaires to provide initial feedback on the training and the slab product. After the village trainings, the sales representatives were responsible for marketing the slabs and facilitating bulk slab purchases in their communities.

#### Text messaging.

For 6 months following the trainings, we sent all 396 sales representatives biweekly text messages using the software Sematime (Sematime, Nairobi, Kenya). The aim of these text messages was to encourage sales representatives to market and sell the slabs. These text messages reinforced the sanitation campaign messages (such as “*Improve your latrine today. Your toilet is your dignity*”) and also specifically promoted the plastic slab (such as “*Improve your toilet with the plastic toilet slab*”). The text messages also included purchasing contact details for the MFI (ECLOF) and slab manufacturer SilAfrica. We provided these text messages in Swahili and the local language of the village (Luhya, Itseo, or Kikuyu), and confirmed message receipt through the Sematime software.

### Data collection.

#### Household surveys.

We conducted baseline surveys in approximately 15 households from each of the 30 study villages (16 Busia and 14 Nyeri) in March 2015 to obtain information on demographics, sanitation facilities and practices, socioeconomic status, water and hygiene practices, and other factors. Two years after baseline, we conducted endline surveys to collect data on exposure to the improved sanitation campaign, sanitation facility improvements, slab purchasing, and perception of the plastic slab product.

#### WTP slab auction.

To determine WTP for the plastic slab, we conducted a real-money auction of the small plastic slab (60 × 60 cm), manufactured by SilAfrica in Nairobi, Kenya. Based on the Becker Degroot Marschak (BDM) auction method,^18^ each household bid its own money for the slab and won the slab if their bid was above a randomly chosen price hidden inside a sealed envelope. Households then paid the price named in the envelope, not their own named price, and both their winning bid and the price paid were recorded in the survey. This BDM method is an incentive-compatible design in which the best strategy for households is to truthfully report their WTP; the auction bid affects whether or not a household wins, but not the actual price paid.

We selected households for the WTP slab auction based on the following criteria: 1) they had not upgraded their sanitation facility with improved flooring and 2) they were not sharing a latrine with another study household already identified as eligible for the auction. Three weeks before conducting the auction, survey team members explained the auction process to all eligible households, showed the plastic latrine slab product, and described the product features. In addition, to ensure that households understood the auction process, they participated in an example auction using a bar of soap. Enumerators also informed households of the slab auction price points (100–1,600 KES in increments of 100 KES [US$1–US$15.7, in increments of just under US$1]), and the market price of the slab of 1,650 KES (US$16.2) (at the time of this study, SilAfrica reduced the price of the plastic slab from 2,500 KES [US$24.5] to 1,650 [US$16.2] to increase demand). Enumerators then notified households that they would return in 3 weeks to conduct the slab auction and left households with a slab brochure specifying their return date. We instructed households to decide on a WTP bid price in collaboration with their other household members and prepare the funds for the amount they were willing to pay.

We revisited households after 3 weeks to conduct slab auctions. If households won the auction, they had until the end of the day to gather sufficient funds. We collected payments as cash or via mobile payments (M-Pesa, Safaricom, Nairobi, Kenya).

#### Willingness-to-pay design comparisons.

**Figure 2.**
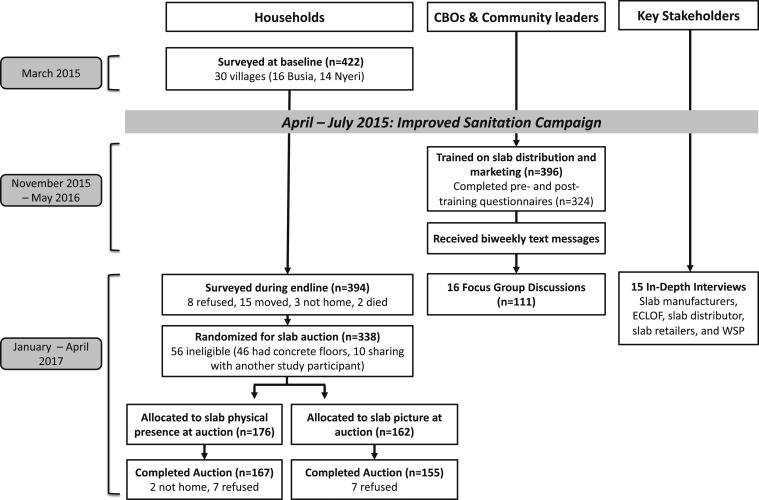
Data collection study flow. CBOs = community-based organizations. We also refer to the CBOs and community leaders as “sales representatives.”

Previous evidence suggests that WTP is higher for products when they are physically present at the time of the purchase decision.^19^ For example, in the rural Kenya context, this may mean having products physically present and available for purchase at the nearest local market (as opposed to having to place an order). To examine whether the physical presence of the plastic slabs increased WTP, we randomized households into two groups for the slab auction: 1) households that had the plastic slab physically present during the slab auction and 2) households that saw a picture of the slab during the auction. All households were shown the physical slab during the endline survey to ensure they were familiar with the product (3 weeks before the auction). Households that won the auction and provided payment were either given the slab immediately (for physical presence group) or by the end of the day (slab picture group). Our sample size of approximately 300 households (30 village clusters with five households each per comparison group) would allow us to detect at least a 15% difference in slab uptake between groups with 90% power, assuming an intra-cluster coefficient of 0.032^20^; our sample size was limited by the number of households recruited for the larger cRCT (Figure 2).

#### Sales representative FGDs and key stakeholder IDIs.

To document slab marketing activities and sales challenges, we conducted FGDs with sales representatives (CBOs and community leaders) and IDIs with key stakeholders, following a semi-structured discussion guide. For FGDs, we randomly selected 16 of the 29 villages with sales representatives (two villages were combined for training). From these selected villages, we invited all individuals who attended the 2015 trainings to attend the FGDs. Two of the coauthors (J. K. and P. R.) conducted all the FGDs in Swahili, with one person acting as the discussion leader and the other as notetaker. To identify key stakeholders for IDIs, we used a “snowball” method,^21^ starting with key contacts of the project (e.g., WSP staff and slab manufacturers). Three coauthors (R. P., J. K., and P. R.) conducted the IDIs in either English or Swahili. For all FGDs and IDIs, we took detailed notes in English and also audio-recorded discussions. Focus group discussions and IDIs were conducted until saturation was reached (i.e., no new information was obtained with additional data collection).

### Data analysis.

We conducted all household surveys using mobile phones (Samsung Galaxy Trend Lite, Seoul, South Korea) using the CommCare survey and data management application (DiMagi Inc., Cambridge, MA). We conducted quality control checks (spot checks and back checks) on approximately 10% of all surveys.

When calculating WTP, households that answered the endline survey but refused to play the slab auction (4%, 14/336) were classified as having a WTP of 0 KES. We classified the participating households into socioeconomic status quintiles using an asset index and principle component analysis. To compare WTP between subgroups, we conducted sample *t*-tests of mean WTP and examined the entire distribution of WTP by performing Kolmogorov–Smirnov tests. An analysis of variance compared mean WTP for categorical variables (e.g., wealth quintiles and education levels). Quantitative data were analyzed using the statistical package Stata 15 (StataCorp, College Station, TX).

For qualitative data analysis, we first supplemented the notes with transcriptions from the audio recordings as necessary. We then analyzed the data for common themes using NVivo software; the coauthors who performed the primary data collection (R. P., P. R., and J. K.) managed the coding and analysis. We triangulated data with findings across the IDIs, FGDs, and household surveys, and also selected illustrative quotations that represented perspectives of most participants.

The exchange rate used for the analysis was 102.0 KES to US$1.00 (May 1, 2017, oanda.com).

### Ethical approval.

All household and FGD participants were informed of the details of the study and provided written consent before study participation; IDI stakeholders provided verbal consent. The study was reviewed and approved by AMREF Ethics and Scientific Review Committee in Kenya (Ref: AMREF-ESRC P155/2014). This study is registered in the AEA RCT Registry at https://www.socialscienceregistry.org/trials/2133/history/16024 with the unique identifying number AEARCTR-0002133.

## RESULTS

We conducted 422 household baseline surveys, 394 household endline surveys (93% of baseline), and 322 household slab auctions (76% of baseline) (Figure 2). Of the 396 sales representatives, 324 (82%) completed pre- and post-training questionnaires. In the 16 villages selected for the FGDs, 111/187 (59%) of the original sales representatives attended the FGDs. We conducted 15 IDIs with key stakeholders.

### Financing and distribution model challenges.

#### Poorly defined stakeholder roles.

From the IDIs and FGDs, we found that the financing and distribution model intervention faced challenges during implementation. Specifically, product marketing and stakeholder engagement were limited because of poorly defined roles and a lack of incentives: many of the program stakeholders (the manufacturers (SilAfrica and Kentainers), the funder (World Bank), the MFI (ECLOF), sales representatives, and government) expected that others would take on more marketing responsibilities to ensure household exposure to the slab product.

#### Sales representatives’ lacked incentives for slab marketing.

Sales representatives performed limited marketing and were demotivated by a lack of compensation for marketing activities and follow-up. Although the program was designed for sales representatives to profit from purchasing the slabs in bulk to then sell to households, no specific funding or compensation was provided to sales representatives for bulk purchases or marketing activities. One sales representative explained, “If we had received incentives it would have been a source of motivation to work harder and market the slabs” (CBO member, FGD 1).

In addition, sales representatives’ effectiveness as marketing agents was compromised by the lack of slab samples to show potential consumers, who were generally unfamiliar with plastic latrine slabs. One sales representative explained, “If you do not have an example, it is difficult to demonstrate the features of the slab. If you have it with you they can step on it and see it does not break easily and it would be easier for me to convince them to buy the slab” (village elder, FGD 1).

Although these sales representatives received biweekly text messages highlighting the importance of improved sanitation and the plastic slab, these were not effective for motivating sales; the sales representatives expected additional follow-up from stakeholders. Sales representatives reported, “After the people who trained us went quiet, I did not follow up so much” (village elder, FGD 1), and “For the first three months, I was active and after lack of communication from the stakeholders, I saw like it was a waste of time marketing the slabs” (mason, FGD 3).

Lastl, masons were not motivated to sell plastic slabs because concrete slab installation was more profitable. “Fundis [masons] would not be able to market the slab because the slab is easy to install and takes few hours hence they are paid less for installation (600 KES [US$5.88] for labor) unlike concrete slabs which take long to install” (mason, FGD 8), and which allows masons to earn 2,500 KES (US$24.5 USD) for their labor. It is also likely that most masons were not familiar with the plastic latrine slab, unless they attended the village trainings.

#### Complicated supply chains for plastic slab purchasing.

The purchasing options for the plastic latrine slabs were complex, requiring cooperation between potential consumers (e.g., purchasing in bulk via SilAfrica or forming a group via ECLOF), or long distance travel to the retail locations in Busia and Nyeri towns. Key stakeholders and sales representatives reported that “buying the slab was a hassle, a real problem” (stakeholder interview) and “the process of acquiring the slab was very complicated” (farmer, FGD 14).

Although the purchasing options were presented in the village trainings, some sales representatives did not know how to purchase the slabs: “I can sacrifice myself and buy it, but [only] if I knew where to find it” (community volunteer, FGD 2) and “I was told where to get the slabs but I forgot the location” (CBO chair, FGD 4). In addition, before the training, only 16% of sales representatives reported hearing of ECLOF, and only 26% had heard of SilAfrica; therefore, a lack of trust discouraged sales representatives to take on new loans through ECLOF or send mobile money payments to SilAfrica before slab delivery, as required for slab purchasing. Although 80% of sales representatives believed that loans were important for purchasing the slabs in the post-training surveys, there were some general concerns of taking on new loans because households “have so many loans” (farmer, FGD 6) and “the institutions that are giving you a loan do not give you time to breathe. They are always following up with you to make sure that you pay back the loan and this prevents people from getting a loan” (farmer, FGD 6).

### Household survey results.

#### Household slab sales and sanitation facilities.

The study household population is described in Table 2 and in a previous report.^13^ At endline, no study households had purchased the plastic latrine slab and almost all (87%, 342/394) still had unimproved latrines. Most latrines (84%, 182/217) in Busia had mud floors, and most latrines in Nyeri (79%, 140/177) had wooden floors (Table 2), similar to baseline.^13^ Nine households (2%, 9/394) did not have a latrine (seven pits collapsed and two households were using neighbors’ latrines) (Table 2).

**Table 2:** Household characteristics

Characteristic	County		
	Busia, *N* = 217	Nyeri, *N* = 177	Total, *N*=394
Gender of respondent			
Male	29% (64/217)	38% (68/177)	34% (132/394)
Female	71% (153/217)	62% (109/177)	66% (262/394)
Education of respondent			
No school	12% (26/217)	8% (15/177)	10% (41/394)
Primary	67% (146/217)	56% (100/177)	62% (246/394)
Secondary	18% (41/217)	32% (57/177)	25% (98/394)
College/university	2% (4/217)	3% (5/177)	2% (9/394)
Wealth Quintile			
Lowest	33% (71/217)	0% (0/177)	18% (71/394)
Low	35% (76/217)	0% (0/177)	19% (76/394)
Middle	32% (69/217)	23% (40/177)	28% (109/394)
High	< 1% (1/217)	41% (73/177)	19% (74/394)
Highest	0% (0/217)	36% (64/177)	16% (64/394)
Type of sanitation (observed)			
No latrine	3% (7/217)	1% (2/177)	2% (9/394)
Latrine with mud floor	84% (182/217)	6% (10/177)	49% (192/394)
Latrine with wood floor	2% (4/217)	79% (140/177)	37% (144/394)
Latrine no or partial slab	< 1% (1/217)	3% (5/177)	2% (6/394)
Latrine with concrete slab	11% (23/217)	11% (20/177)	11% (43/394)
Latrine usage while at home			
Everybody	96% (201/210)	93% (163/175)	95% (364/385)
Not children aged < 5 years	3% (7/210)	7% (12/175)	5% (19/385)
Not children aged 5–10 years	1% (2/210)	0% (0/175)	< 1% (2/385)
Made latrine improvements in the past 2 years			
Yes	43% (94/217)	46% (81/177)	44% (175/394)
No, but planned	48% (105/217)	38% (68/177)	44% (173/394)
No	8% (18/217)	16% (28/177)	12% (46/394)
Observed sanitation activities in the community in the past 2 years			
None	–	23% (41/177)	–
New latrines in the community	7% (16/217)	41% (73/177)	14% (57/394)
New latrines in schools	68% (147/217)	32% (56/177)	55% (220/394)
Radio show promotion	61% (132/217)	27% (48/177)	48% (188/394)
Sanitation community meetings	64% (138/217)	17% (30/177)	47% (186/394)
Road shows with sanitation messages/products	51% (111/217)	3% (6/177)	36% (141/394)
Community-led total sanitation triggering event	10% (21/217)	2% (4/177)	7% (27/394)
Other*	8% (17/217)	3% (5/177)	5% (21/394)
Do not know	1% (3/217)	2% (3/177)	2% (8/394)
	< 1% (1/217)	–	1% (4/394)
Exposure to sanitation improvement messaging in the past 2 years†			
No	67% (146/217)	81% (141/175)	73% (287/392)
Yes, community health volunteer (CHV)	23% (55/217)	14% (24/175)	20% (79/392)
Yes, other‡	8% (17/217)	5% (9/175)	7% (26/392)
Do not know	0% (0/217)	1% (2/175)	< 1% (2/392)
Exposure to plastic latrine slab			
Not heard of or seen slab	36% (79/217)	31% (54/177)	34% (133/394)
Heard of a slab, but not seen a photo or a physical slab	17% (37/217)	20% (36/177)	19% (73/394)
Heard of a slab, and seen a photo	29% (63/217)	33% (59/177)	31% (122/394)
Heard of and seen a physical slab	18% (38/217)	16% (28/177)	17% (66/394)

* Other = funeral (3), television (1), church (1), other home visits (3).

† Households were asked if anyone had spoken to them about improving their sanitation in the past 2 years.

‡ Other = public health officers (8), participated in village training (5), relatives (3), village chiefs (3), church members (3), ECLOF (2), research study staff (2), received text message as part of the study (1).

Despite the absence of plastic slab purchases by the study endline, 11% (43/394) of households had a latrine with a concrete slab, indicating they had made upgrades to their latrines. Of households that upgraded their latrines, average (median) spending was 14,400 KES (US$141, interquartile range: 5,800–30,000 KES [US$57–294]). This spending generally included other latrine improvements in addition to concrete slab installation, such as improving ventilation, repairing or rebuilding the superstructure, and/or building a new door, roof, or walls.

#### Exposure to improved sanitation campaign.

Regarding exposure to the improved sanitation campaign, 47% (186/394) of the households reported hearing the radio show promotion, but only 7% (27/394) were familiar with the road shows. Within the past 2 years, 27% (105/394) of households had received some form of exposure to sanitation improvement messaging, most commonly (20%, 79/392) through CHVs (Table 2). In addition, 86% (337/394) had observed some form of sanitation improvement activities in their communities in the past 2 years (Table 2). We were unable to determine the extent to which these improvement activities were a direct result of the improved sanitation campaign.

#### Exposure to and perceptions of the plastic latrine slab.

We found that most households had limited exposure to the plastic slabs. At endline, 83% (328/394) of households had never seen the physical slab and 34% (133/394) had never heard of or seen the slabs at all (Table 2). The primary exposure to the plastic slabs was when enumerators showed households a photo of the plastic slab during baseline data collection; 56% (147/261) of households that were familiar with the slab reported this method of exposure. During the endline survey, most households (276/394, 70%) reported that their main reason for not purchasing the slab was because they were unfamiliar with the product.

Almost all households (99%, 258/261) that were familiar with the plastic slab reported that they liked the product. When asked to rate various characteristics of the slab, more than 90% of households agreed that the slab looked good, was easy to clean, was easy to use, felt sturdy, prevented smells, and was lightweight. Sales representatives also had positive perceptions of the slab; post-training questionnaires indicated that 96% (311/324) liked the slab and 96% (312/324) believed people would purchase the slab. During FGDs, sales representatives reported that the slab was “good,” “long lasting,” “easy to use” for adults and children, “has a lid that prevents flies,” portable, and modern (FGD 7). During the FGDs and IDIs, there was some feedback that the slab products were too small; however, most sales representatives and households had positive opinions of the slab size, with 90% (292/324) of sales representatives reporting that the size was appropriate in the post-training questionnaire.

### Slab affordability and demand.

#### Slab affordability perceptions.

Many households and sales representatives perceived the slab to be unaffordable: only 24% (62/261) of households familiar with the slab at endline rated the product to be at a good price. Some stakeholders reported that the slab was not “value for money,” particularly compared with prices of other plastic products, and believed the price was too high: “even myself in my heart, I thought it was too expensive” (village elder, FGD 4). Sales representatives also reported that “most people have more pressing priorities” than buying a slab (FGD 14).

#### Slab auction results.

Overall, 47% (151/322) of households won the auction and 91% (137/151) of the auction winners purchased the slabs; of the remaining, 12 reported they did not have the money and two could not make the decision to purchase without additional consultation. Most households (88%, 120/137) paid for the slabs with cash, 11% (15/137) paid via M-Pesa, and 1% (2/137) used a combination of cash and M-Pesa.

**Figure 3.**
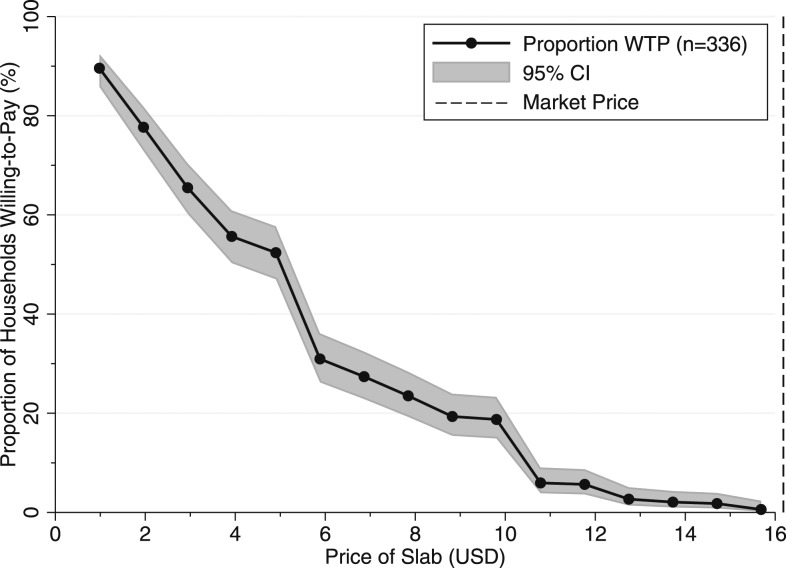
Willingness to pay (WTP) for the plastic latrine slabs. US$1 = 102 KES. The solid line is the proportion of households WTP and the gray area represents the 95% CI. The market price of the slabs is US$16.2, represented by the dashed line. The median WTP was US$5.

Almost 90% (301/336) of households bid on the product (i.e., WTP > 0 KES; 14 households refused to play and 21 bid 0 KES). Half (176/336) of respondents were willing to pay at least 500 KES (US$4.9) for the plastic slab and almost 20% (63/336) of respondents were willing to pay 1,000 KES (US$9.8) (Figure 3). However, less than 1% (2/336) of households were willing to pay 1,600 KES (US$15.7), the approximate market price of the slab at the time of this study. The mean WTP was 480 KES (US$4.7) and the median was 500 KES (US$4.9) (Table 3).

**Table 3:** Mean and median WTP for subgroup analyses

Subgroup	*N*	Mean WTP (KES)	Median WTP (KES)	Interquartile range*	*P*-value (*t*-test of means)†
County					0.99
Busia	190	480	400	200–700	
Nyeri	146	479	500	200–700	
Gender					0.21
Male	110	516	500	200–700	
Female	226	462	400	200–700	
Wealth quintile					0.91
Lowest	67	452	300	200–700	
Low	65	477	400	200–600	
Middle	93	480	500	200–800	
High	64	517	500	200–1,000	
Highest	47	470	500	200–500	
Treatment					0.98
Picture	162	479	500	200–700	
Physical slab	174	480	500	200–700	
Education					0.77
None	33	497	400	300–800	
Primary	215	469	500	200–600	
Secondary or beyond	88	500	500	200–750	
Age (years)					0.12
0–49	195	508	500	200–800	
50+	139	444	400	200–600	
Exposure to sanitation improvement messaging in the past 2 years					0.11
No	245	499	500	200–800	
Yes	89	426	400	100–500	
Sanitation activities in the past 2 years					0.88
Not observed	50	472	500	200–700	
Observed in community	282	480	500	200–700	
Made latrine improvements in the past 2 years					0.53
No	190	497	500	200–700	
Yes	139	471	500	100–700	
Slab exposure/familiarity					0.05
Not seen a photo or a physical slab	179	443	400	100–600	
Seen a photo or a physical slab	157	522	500	200–800	
Plan to install a slab‡					0.04
No	168	464	450	200–700	
Yes	29	614	500	300–1,000	
Have a M-Pesa account§					< 0.01
No	27	289	100	100–500	
Yes	305	495	500	200–700	
Have children aged < 5 years‖					0.84
None	160	485	500	200–700	
Kids aged < 5 years	175	477	500	200-700	
Reported treating water‖					0.62
No	94	496	500	200–700	
Yes	242	473	500	200-700	
Reported sharing latrine‖					0.12
No	215	504	500	200–800	
Yes	120	438	350	100–600	
Reported taking out a loan or borrowing money to build their latrine§					0.05
No	306	470	500	200–700	
Yes	20	635	500	500–900	
Reported receiving subsidy to build their latrine‖					0.59
No	309	480	500	200–700	
Yes	15	533	600	200–1,000	
Total population	336	480	500	200–700	

WTP = willingness to pay.

* Interquartile range = 25th to 75th percentile.

† Two-sided sample *t*-test was used to compare means; analysis of variance was used to compare for wealth quintiles and education.

‡ Only households that had not upgraded their latrine within the past 2 years were asked about plans to install a slab (*n* = 197).

§ M-Pesa is a mobile phone payment system.

‖ These characteristics were reported at baseline.

We did not find any significant difference in WTP by county, gender, wealth quintile, or treatment (i.e., seeing slab picture versus physical slab during auction) (two-sided *t*-test and Kolmogorov–Smirnov tests, all *P* > 0.05, Table 3, Supplemental Figure 1). When examining associations with other characteristics, we found that WTP was significantly higher among households that had a M-Pesa account (two-sided *t*-test, *P* < 0.01) and households that reported plans to install a slab (two-sided *t*-test, *P* = 0.04). Willingness to pay was borderline significantly associated with being familiar with the plastic slab (*P* = 0.054) and taking out a loan or borrowing money for latrine construction (*P* = 0.055).

## DISCUSSION

This is the first study to examine plastic latrine slab uptake in rural households in Kenya. We found that the primary barriers to slab sales were 1) insufficient marketing activities, which led to limited household exposure to the slab product, and 2) low demand for the slab product at the specified sales price. Product marketing was limited because of a lack of incentives for stakeholders; the manufacturers and MFI (ECLOF) had other products that were more profitable, and the sales representatives were provided neither with compensation, slab samples, nor follow-up support. Similarly, stakeholders had minimal incentives to simplify distribution channels, resulting in complicated purchasing options for households. These barriers raise questions about the viability of charging unsubsidized prices for preventive health products that additionally face last-mile challenges to serving poor consumers in remote, rural settings.

Because of limited marketing, most households were not familiar with the slab product and none of the study households had purchased the plastic latrine slab. Nevertheless, 11% of households had upgraded their unimproved latrines to improved ones by installing a concrete slab. It was not possible to determine whether this increase was a result of the national sanitation campaign, additional sanitation messaging due to the study, or other factors. This 11% proportion is comparable to a recent systematic review that found that sanitation education interventions increased latrine coverage by 14% on average.^22^

Among those familiar with the product, perceptions of the slab product were largely positive. Our WTP auction showed that household interest in the plastic latrine slabs was high: almost 90% of households bid on the product, and half were willing to pay 500 KES (US$4.9). As the product approached market price (1,600 KES [US$15.7]), however, purchase rates dropped to less than 1%. These results are comparable to our previous measurements of WTP for latrine slab products in Tanzania: in a randomized, voucher-based real-money sales trial, which we conducted in Tanzania in 2015, we evaluated demand for the same plastic latrine slab (60 × 60 cm, manufactured by SilAfrica), and two other latrine slab products (cement slabs and ceramic pour-flush slabs). We found that 60% of rural Tanzanian households were willing to pay approximately US$1 for a plastic slab (compared with 90% in Kenya), and only 5% of Tanzanian households were willing to pay US$12 (compared with 6% in Kenya).^20^

The low WTP should be interpreted in the rural Kenyan context. For example, initial slab prices were approximately 30–60% of Kenya’s median monthly income monthly (estimated to be US$66 in 2015).^23^ Households have many competing spending priorities, including some that cannot be postponed, such as paying back other loans, school fees, farming expenses such as seeds, or medical expenses. In addition, at the time of the 2017 FDGs, households in Kenya were facing a drought that increased food prices and limited food availability.^24^

The physical presence of the slab during the auction did not influence WTP, suggesting that the slabs do not necessarily have to be physically present to ensure sales. Similarly, in Tanzania, we found that the physical presence of the slab within the village (i.e., slabs installed in two randomly selected households within the village) did not influence household demand.^20^ However, our qualitative research findings in Kenya indicate that households are unlikely to purchase the slab when they are unfamiliar with the product, and additional strategies are needed to improve slab familiarity.

Household WTP was significantly higher among households that had a mobile money (M-Pesa) account. Households with M-Pesa may have easier access to cash because M-Pesa facilitates money transfers between users.^25^ M-Pesa usage is widespread in Kenya, with more than 90% of our study households having M-Pesa accounts (and more than 85% of households in each of our study wealth quintiles). However, most households that won the auction used cash to purchase the latrine slab.

This study is not without limitations. First, in collecting perceptions of the plastic slabs, there was the possibility of courtesy bias; we were unable to verify whether the positive feedback on the plastic slab product was genuine, or whether respondents were overstating their satisfaction because of a fear of offending the interviewer. Furthermore, the positive feedback could also be the consequence of households and sales representatives having limited familiarity with the slab. However, our WTP assessment using a real-money auction validated household interest in the plastic slab, with almost 90% of households bidding on the product; this result indicates that courtesy bias was not a limitation of the study. Second, it is possible that not all households understood the auction (4% of households won the auction but did not purchase the slab); however, we did conduct example soap auctions with all households so that they were familiar with the process. Third, it is possible that WTP may have been higher if households had a longer time period to save money (i.e., more than the 3 weeks given in the study between the introduction and conduction of the auction). Fourth, because price was the main high barrier, we were unable to determine the extent to which the limited marketing and complicated purchasing options influenced consumer demand. Similarly, we were unable to specifically examine WTP of the sanitation campaign because it was not possible to distinguish the sanitation campaign from other sanitation messaging and improvement activities in the community. Last, most of our respondents were female (71%) and may not always be in charge of household financial decisions; however, there is increasing evidence that Kenyan women are involved in household decision-making,^26^ and we found that WTP did not vary significantly by gender.

Our findings indicate that the current household demand for the plastic latrine slabs is too low to support commercial distribution. Despite the investments made in plastic latrine slab development and marketing interventions, including the improved sanitation campaign, the plastic slabs have not experienced the market growth predicted by the Selling Sanitation Program. The plastic slab manufacturers planned to recover their investment costs through a large volume of slab sales; however, sales have been virtually non-existent to date. Presently, Kentainers continues to sell plastic slabs to international nongovernment organizations, and SilAfrica is now partnering with Lixil to manufacture a smaller plastic latrine pan, the SATO toilet which costs 500 KES (US$4.9).^27^

It is unlikely that further demand creation activities will substantially increase consumer WTP among this population in Kenya. Our current WTP data are for households that were already exposed to a sanitation demand campaign, and we did not find increases in WTP for households that had been exposed to sanitation messaging or had observed recent sanitation activities (Table 3). These findings are in-line with previous research in Tanzania that found demand creation efforts did not increase household WTP: a randomized, controlled trial of a sanitation campaign found that the campaign did not increase household spending on sanitation improvements.^28^

To leverage the substantial investments made into the Selling Sanitation project and plastic slab product development, further efforts are needed to align product prices with consumer WTP. Longer term public private collaboration could support activities such as increasing slab marketing by providing incentives for stakeholders, simplifying purchasing options for households by learning from efforts to date,^16,29^ and lowering the cost of the plastic slab through partial subsidies. Although national policy in Kenya has previously discouraged sanitation subsidies, the Kenya Environmental Sanitation and Hygiene Policy 2016–2030 acknowledges that subsidies may be necessary to ensure adequate sanitation for poor and marginalized populations.^10^ Furthermore, a recent evaluation of strategies to improve sanitation in rural Bangladesh found that household subsidies (using household vouchers) were an effective intervention for increasing hygienic latrine ownership.^30^ Similarly, an assessment of six case studies on sanitation financing found that partial public funding could increase household sanitation by 20–70%.^31^ Other studies have demonstrated that subsidies effectively promote the purchase of other essential health products by poor households.^32–35^

However, to justify additional funding on the plastic latrine slabs, evidence is needed for their reduced pathogen exposure for users and subsequent health benefits. To date, there is little evidence of the incremental health effects of improved sanitation facilities.^36^ Specifically, a large cluster-randomized trial (WASH Benefits) found that a sanitation intervention including plastic latrine slabs did not reduce child diarrhea or improve child growth in Kenya^37^; however, other sanitation upgrades did detect child diarrhea reductions in Bangladesh.^38^ Last, given that other studies have found that households may prefer building new latrines to simple upgrades,^39,40^ other improved sanitation solutions should also be considered.

## Supplemental materials

Supplemental figure
