# Supplementary figures and images for: Assessing the Demand for Plastic Latrine Slabs in Rural Kenya
Source: Am J Trop Med Hyg. 2019 Aug 5;101(3):555–65. doi: 10.4269/ajtmh.18-0888 (PMC6726948; doi:10.4269/ajtmh.18-0888)

## Supplementary info

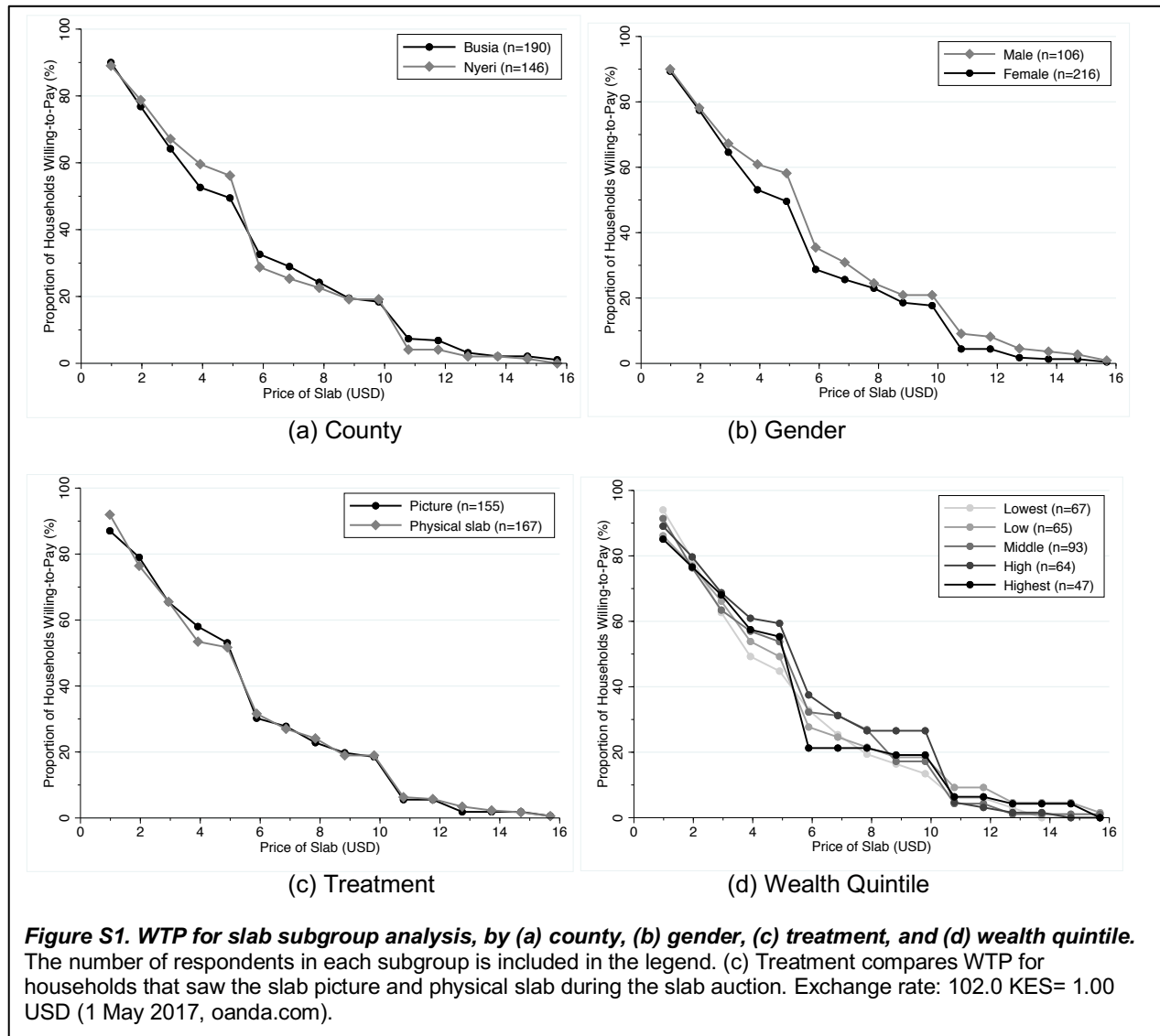

Supplement: Supplementary file 1 [file tpmd180888.SD1.pdf]
